# Genomic sequence and analysis of a vaccinia virus isolate from a patient with a smallpox vaccine-related complication

**DOI:** 10.1186/1743-422X-3-88

**Published:** 2006-10-25

**Authors:** Guiyun Li, Nanhai Chen, Zehua Feng, R Mark L Buller, John Osborne, Tiara Harms, Inger Damon, Chris Upton, David J Esteban

**Affiliations:** 1 Department of Biochemistry and Microbiology, University of Victoria, Victoria, Canada; 2 Department of Molecular Microbiology and Immunology, St. Louis University, St. Louis, USA; 3 Centers for Disease Control and Prevention, National Center for Infectious Diseases, Atlanta, USA

## Abstract

**Background:**

Vaccinia virus (VACV)-DUKE was isolated from a lesion on a 54 year old female who presented to a doctor at the Duke University Medical Center. She was diagnosed with progressive vaccinia and treated with vaccinia immune globulin. The availability of the VACV-DUKE genome sequence permits a first time genomic comparison of a VACV isolate associated with a smallpox vaccine complication with the sequence of culture-derived clonal isolates of the Dryvax vaccine.

**Results:**

This study showed that VACV-DUKE is most similar to VACV-ACAM2000 and CLONE3, two VACV clones isolated from the Dryvax^® ^vaccine stock confirming VACV-DUKE as an isolate from Dryvax^®^. However, VACV-DUKE is unique because it is, to date, the only Dryvax^® ^clone isolated from a patient experiencing a vaccine-associated complication. The 199,960 bp VACV-DUKE genome encodes 225 open reading frames, including 178 intact genes and 47 gene fragments. Between VACV-DUKE and the other Dryvax^® ^isolates, the major genomic differences are in fragmentation of the ankyrin-like, and kelch-like genes, presence of a full-length Interferon-α/β receptor gene, and the absence of a duplication of 12 ORFs in the inverted terminal repeat. Excluding this region, the DNA sequence of VACV-DUKE differs from the other two Dryvax^® ^isolates by less than 0.4%. DNA sequencing also indicated that there was little heterogeneity in the sample, supporting the hypothesis that virus from an individual lesion is clonal in origin despite the fact that the vaccine is a mixed population.

**Conclusion:**

Virus in lesions that result from progressive vaccinia following vaccination with Dryvax are likely clonal in origin. The genomic sequence of VACV-DUKE is overall very similar to that of Dryvax^® ^cell culture-derived clonal isolates. Furthermore, with the sequences of multiple clones from Dryvax^® ^we can begin to appreciate the diversity of the viral population in the smallpox vaccine.

## Background

The highly lethal disease smallpox is caused by Variola virus (VARV), a member of the *Orthopoxvirus *(OPV) genus of the family *Poxviridae*. Smallpox was declared eradicated in 1980 following a global vaccination program launched by the World Health Organization (WHO) [1]. Dryvax^®^, the smallpox vaccine used in the US, played a key role in this global vaccination campaign. Dryvax^® ^was produced by collecting the lymph from bovine calves or sheep following skin scarification with Vaccinia virus (VACV), and isolating the virus, a procedure that is no longer approved by the US Food and Drug Administration (FDA). This produced a mixed population of viruses since no attempt was made to clonally purify the inoculum. By today's standards, Dryvax^® ^has a high incidence of unacceptable side effects, especially considering the current low risk of VARV infection that could only result from an accidental release from the repositories at the Centers for Disease Control and Prevention (CDC, Atlanta, GA) or Vector Institute (Russia), or intentional release as a biological weapon. However, as a result of the immunogenic cross-reactivity characteristic of the OPVs, Dryvax^® ^is also useful as a vaccine against monkeypox virus (MPXV), which causes a smallpox-like disease in humans but with a lower mortality rate and limited human-to-human transmission [2-4]. A recent outbreak of MPXV in the Midwestern U.S.A. forced world health authorities to recognize that this disease is not limited to remote areas of Africa [5,6].

Although a limited quantity of aging stocks of Dryvax^® ^appeared efficacious in recent clinical trials [7,8], they were insufficient to vaccinate the majority of the American population. Therefore a new smallpox vaccine derived from Dryvax^® ^is being manufactured to meet current FDA regulations for potency and composition using tissue culture cell methodology. Because Dryvax^® ^contains a mixture of viruses with different virulence characteristics [9], the new vaccine was based on the isolation and comparison of clones isolated from Dryvax^®^. VACV-ACAM2000 and VACV-CLONE3 are two of six clones isolated from Dryvax^® ^which have been fully sequenced and compared in animal models of infection [10,11]. In animal infection models, VACV-CLONE3 was shown to be more virulent than VACV-ACAM2000 and Dryvax^® ^[10,11] and consequently VACV-ACAM2000 was selected for further trials with the hope that it would be licensed as a second-generation smallpox vaccine. Although phase II trials showed no significant differences in side effects between VACV-ACAM2000 and Dryvax^® ^[12], myopericarditis (swelling of the heart muscle) was reported in the third stage of clinic trials of VACV-ACAM2000, and the trial was halted [13].

Vaccinia virus VACV-DUKE was isolated in December 1970 from a 54-year-old female patient in the eastern US diagnosed with vaccinia necrosum. The virus is thought to have been isolated from a single skin crusted lesion, but the source may have been as many as four lesions. In this study, we present the sequence of the complete coding region of the VACV-DUKE genome. Although other clones derived from Dryvax^® ^have been sequenced, VACV-DUKE is unique in being the only completely sequenced Dryvax^® ^isolate obtained from a human source. Detailed analysis of its nucleotide sequence and gene content contribute to our knowledge of the population diversity of Dryvax^®^, and the lack of sequence heterogeneity suggests a single clonal lesion source.

## Results

### Overview

The 199,960 bp VACV-DUKE coding region was assembled from 1891 readings of 441 bp average length and 4.17-fold redundancy. Like other OPVs, VACV-DUKE is AT rich (66.6%) and the genome can be divided into a central region of 174,892 bp, and two inverted terminal repeat (ITR) regions of 12,534 bp at each end of the genome (1-12,534, 187,427-199,960). There are two direct repeats (DR) in each ITR (DR1 and DR2 in the left ITR, DR3 and DR4 in the right ITR). DR1/DR4 has a calculated 19 copies of a 70 bp tandem repeat element with a single nucleotide variation in different copies: the 1^st^, 2^nd^, 3^rd^, and 5^th ^copy of DR1/DR4 lack the 42^nd ^A of the repeat element. However, the exact sequence of all copies is not known; since the repeat region was too large to sequence through in two opposing reads, only 6 of the 19 copies have been fully sequenced. This tandem repeat element is identical to the 70 bp tandem repeat in VACV-ACAM2000 and VACV-CLONE3. DR2/DR3 contains 9 copies of a 54-bp tandem repeat element that is also identical to the 54 bp tandem repeat element found in VACV-ACAM2000 and VACV-CLONE3.

**Figure 1:**
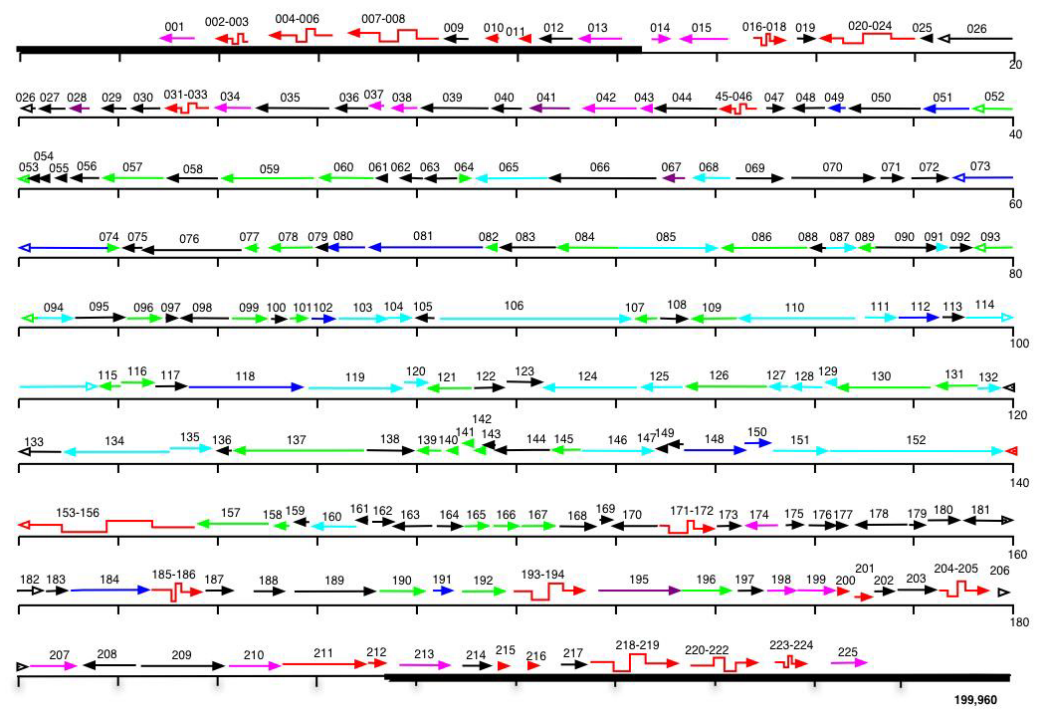
**Genome map of VACV-DUKE**. Predicted genes are numbered and represented by colored arrows (green: structure and assembly; light blue: RNA metabolism; dark blue: DNA metabolism; dark purple: host range; pink: immunomodulators or virulence factors; black: unknown function; red staggered: fragmented orthologs of ORFs present in other OPVs; red straight arrow: truncated orthologs of ORFs present in other OPVs). Open arrowheads indicate an ORF is split over 2 lines of the diagram. Scale is shown in kb. Thick black lines represent the ITRs.

225 non-overlapping ORFs were initially detected in VACV-DUKE. Comparative analyses with other poxviruses resulted in the assignment of these ORFs to 178 full-length, probably functional genes, and 47 gene fragments that most likely do not direct the expression of functional gene products (Figure 1) [also see Additional file 1]. Similar to other OPVs, the genes are located on both strands of the linear dsDNA genome with very small intergenic regions, and the majority of genes present within 50 kb of each terminus are transcribed towards that end of the genome. Three full-length genes and 5 fragmented regions are duplicated in the ITRs [see Additional file 1]. In addition, the Serine Protease Inhibitor (SPI) gene in the right ITR (ORF 213) is partially duplicated in the left ITR (ORF 13). However, it is not obvious if the deletion of the 5' end of ORF 213 results in a truncated protein because the actual translational start site for the gene is not known. VACV-DUKE possesses all 49 genes that comprise the minimum essential genome conserved among all completely sequenced poxviruses and the 89 genes conserved in all OPVs [14].

### Phylogenetic analysis

**Figure 2:**
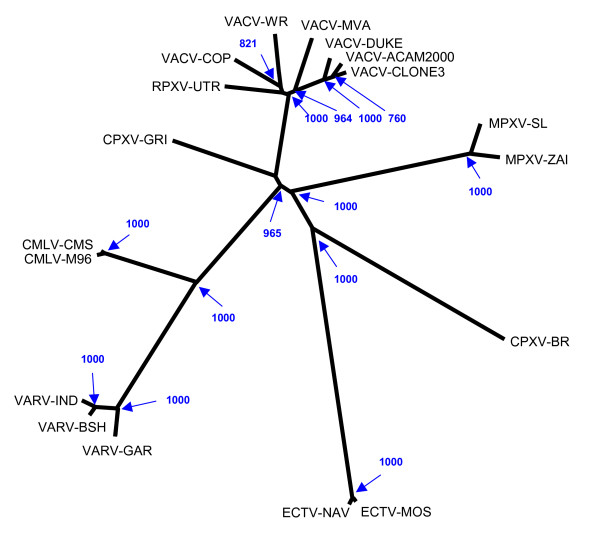
**Phylogenetic tree of the orthopoxviruses**. The tree was generated using an 82 kb DNA sequence conserved among all fully sequenced OPVs. Sampling number for bootstrap is 1000; only bootstrap values above 500 are shown.

To confirm the origin of VACV-DUKE as an isolate of Dryvax^®^, we performed phylogenetic analysis using the central 82 kb (nt 58159 to nt 139791 of VACV-DUKE) that is present in all completely sequenced OPVs. In this region at the nucleotide level VACV-DUKE is most similar to VACV-ACAM2000 (99.75% identical) and most distant from Ectromelia virus (ECTV; 97.46%). The phylogenetic tree (Figure 2) clearly indicates that VACV-DUKE is closely related to other Dryvax^® ^isolates.

### Comparison of VACV-DUKE, VACV-CLONE3 and VACV-ACAM2000

**Figure 3:**
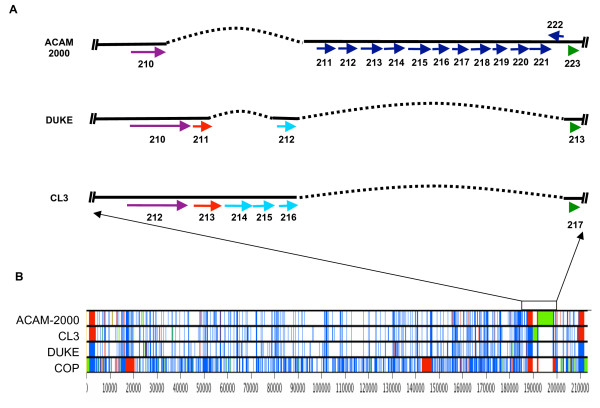
**Map of genomic differences among DUKE, CLONE3 and ACAM2000**. A) Map of the region of major genomic differences among the three Dryvax^® ^clones Solid black line: viral genome; dashed black line: deletion. ORFs are shown as colored arrows. Purple: IFN-α/β receptor homolog which is intact in DUKE and CLONE3 but truncated 90 amino acids from the C terminus in ACAM2000; Red: Ankyrin-like gene, which is intact in CLONE3, fragmented in DUKE and absent from ACAM2000; Light blue: fragments of Kelch-like gene; Dark blue: genes present in the right ITR of ACAM2000 due to a duplication; Green: Serpin (SPI) gene. Gene lengths are not drawn to scale. B) Overview of the positions of substitutions (blue bars), insertions (green bars) and deletions (red bars) among the genomes of three Dryvax^® ^clones, and VACV-Cop, a VACV strain not derived from Dryvax^®^. Substitutions, insertions and deletions are relative to the consensus sequence. Genomes were aligned using Clustal W and edited in Base-By-Base. Numbers indicate the nucleotide position.

We analyzed both the gross and minor differences between these three Dryvax^® ^isolates to survey the diversity of the vaccine preparation. An alignment of the complete coding regions of VACV-CLONE3, VACV-ACAM2000 (GenBank accession AY313848 and AY313847, and Joe Esposito, personal communication) and VACV-DUKE demonstrates that they are highly conserved except in the region from VACV-DUKE-nt 18,4997 to nt 18,7423 (Figure 3). The interferon (IFN)-α/β receptor gene (ORF 210) is complete in VACV-DUKE and VACV-CLONE3, but is truncated in VACV-ACAM2000 as a result of a deletion of approximately 4 kb compared to VACV-CLONE3 [see Additional file 1] (Figure 3). The ankyrin-like gene (ORF 211; ortholog of VARV-BSH-B18R) is complete in VACV-CLONE3 but truncated in VACV-DUKE and absent from VACV-ACAM2000. The truncation in VACV-DUKE is the result of a deletion of approximately 2 kb relative to VACV-CLONE3 that also deletes a portion of the kelch-like gene (ORF 212). The kelch-like gene, which is complete only in ECTV and CPXV, is fragmented into 3 pieces in VACV-CLONE3, and only the third part is present in VACV-DUKE. Finally, 12 genes and gene fragments are duplicated in the left and right ITRs of VACV-ACAM2000 whereas they are present as a single copy in the left ITR of VACV-DUKE and VACV-CLONE3.

Other major gene differences are present [see Additional file 1]. ORF 188 in VACV-CLONE3, (Unknown function, VACV-COP-A51R ortholog), is fragmented in VACV-DUKE (ORFs 185 and 186) and in VACV-ACAM2000 (ORFs 185 and 186) as a result of a 4 bp deletion that causes a frameshift. In addition to the IFN-α/β receptor, VACV-DUKE has two other genes that are full length but are fragmented in at least one other Dryvax^® ^isolate: the ortholog of VACV-DUKE-ORF 39 (Ankyrin, VACV-COP-M1L ortholog) is fragmented in VACV-CLONE3 as a result of a frameshift caused by a 2 bp deletion, and the ortholog of VACV-DUKE-ORF 69 (virosome component) is fragmented in both VACV-CLONE3 and VACV-ACAM2000 as a result of an 11 bp deletion resulting in a frameshift. VACV-DUKE-ORF 177 (Unknown function, MVA-156R ortholog) is identical in VACV-CLONE3 but deleted in VACV-ACAM2000. VACV-ACAM2000 ORFs 195 and 196 are fragments of the ECTV-MOS-153 ortholog, however, VACV-ACAM2000 ORF 196 is 140 and 142 amino acids shorter than the orthologs in VACV-DUKE (ORF 194) and VACV-CLONE3 (ORF 196), respectively. Finally, VACV-ACAM2000 encodes 3 proteins that compared to VACV-DUKE and VACV-CLONE3, are truncated by at least 20 amino acids [thymidylate kinase (DUKE ORF-182), tumor necrosis factor (TNF)-α receptor homolog (DUKE ORF 188), and the ortholog of VACV-COP-B11R (DUKE ORF 202)].

On the nucleotide level, there are a total of 689 insertions/deletions (indels) and substitutions between VACV-DUKE and VACV-CLONE3, and 664 between VACV-DUKE and VACV-ACAM2000 (Figure 3B). Although there are more indels and substitutions in coding regions between VACV-DUKE and VACV-ACAM2000 (534) than between VACV-DUKE and VACV-CLONE3 (495), these result in only a total of 182 and 180 amino acid changes, respectively, indicating that the proteomes of the three viruses are highly similar. The largest amino acid difference between a single full-length ORF is between VACV-DUKE ORF 19 and VACV-CLONE3-ORF 19, the IL-18 binding protein, with 89.5% identity. For comparison, there are 892 indels and more than 1000 substitutions between VACV-DUKE and VACV-COP. Comparing only the 174 shared full-length gene sequences, VACV-DUKE and COP have lower identity (98.84%) than VACV-DUKE and VACV-CLONE3 (99.49%) or VACV-DUKE and VACV-ACAM2000 (99.59%), showing the three Dryvax^® ^isolates are more closely related.

The ankyrin-like gene (VARV-BSH-B18R ortholog) is intact in VACV-CLONE3 but encodes only a 242 amino acid C-terminal fragment in VACV-DUKE (ORF 211) and is totally absent from VACV-ACAM2000. This ORF is intact in CPXVs, VARVs, MPXVs, RPXV (encoding 783–800 amino acid proteins), and truncated in other OPVs (650–661amino acids). The function of this protein is unknown, however, some features are apparent from its sequence. As in many ankyrin-repeat containing proteins in poxviruses there is an F-box motif in the C terminus that has been shown in other proteins to play a role in the ubiquitination pathway [15]. Although the F-box is not conserved in VACV-DUKE, the remaining fragment encoding the ankyrin repeat is intact. There are numerous ankyrin repeat-containing proteins in poxviruses, only a few of which have been explored for function [16,17].

ORF 212 (kelch-like gene) is full length in CPXV and ECTV (ECTV-MOS-167) and fragmented into three pieces in VACV-CLONE3, only the third of which is present in VACV-DUKE. This fragment is also present in VACV-WR and MPXV. The gene is absent from VACV-ACAM2000, and fragmented or absent from other OPVs. The function of this gene is unknown, and the conserved fragment does not contain a kelch-like domain (only the first two fragments have this domain). The fragmented gene is not expected to be functional.

## Discussion

VACV-DUKE is unique in that it was recovered from a patient suffering from a vaccine-related complication. It is not known if the patient was vaccinated, or became infected following contact with a vaccinated individual. The immune status of the individual is not known, thus the underlying basis for the clinical findings is not clear. It is possible that the patient was immunosuppressed and vaccination resulted in dissemination of the virus, and disease. Furthermore, because virus was isolated from only one or a few lesions it is not known how the sequence of VACV-DUKE would compare to virus isolated from other lesions. This could provide insight into the properties of the viruses that disseminate and cause disease in vaccine-related complications. The method used for sequencing (sequencing of PCR products generated directly from genomic DNA) without plaque purification of virus, yielded a single genomic sequence, indicating that a single genome is present in the sample, or that if other genome sequences are present, they are found at a frequency too low to be captured by the initial PCR and sequencing reaction. Because of this lack of heterogeneity in the data, this study suggests that an individual lesion is the result of replication of a single clone from the mixed population of virus delivered at the inoculation site. Although this result is not surprising, it has not been previously formally demonstrated. In addition, genomic characterization of this virus permits further analysis of the population diversity in Dryvax^® ^and indicates that both nucleotide level and larger genomic differences exist in the vaccine.

In addition to host factors such as immune status, vaccine side effects may be due to specific, more pathogenic, virus genotypes present in the mixed population, or a result of a combination of multiple genotypes. Differences in pathogenicity between VACV isolates could be attributed to the presence or absence of a single gene, multiple genes, point mutations within genes or even changes in promoter sequences, causing differences in expression levels of virulence factors. The virulence of VACV-DUKE has not yet been tested in mice, and it is not clear, based on the limited information available about the patient from which it was isolated, how virulent it is likely to be in humans. It will be necessary to perform further experiments to determine the virulence of VACV-DUKE in animals to form a better understanding of the contribution of small or large genomic differences between viruses to pathogenicity.

The most striking region of difference between the genomes of the three Dryvax^® ^isolates occurs near the right ITR. Three genes are affected: the IFN-α/β receptor gene, the ankyrin (VARV-BSH-B18R) homolog, and the kelch-like gene, ORF 212. The IFN-α/β receptor has three immunoglobulin domains that bind host IFN-α/β with high affinity and broad species specificity, although binding affinity for human IFN-α is significantly greater than for mouse IFN-α [18]. While both VACV-DUKE and VACV-CLONE3 have an intact IFN-α/β receptor gene, a DNA deletion in VACV-ACAM2000 results in a C-terminal 90 amino acid truncation, effectively eliminating the third immunoglobulin domain. It is expected that the VACV-ACAM2000 expressed protein would bind the ligand with reduced affinity similar to the ortholog expressed by VACV-Wyeth that also has a truncated C-terminal immunoglobulin domain and binds IFN with lower affinity [18]. Deletion of the IFN-α/β receptor homolog in VACV-WR results in significant attenuation in an intranasal model of infection in mice [19]. Because of its demonstrated role in virulence, it is possible that the presence of the full length gene in VACV-CLONE3 contributes to its greater pathogenicity compared to VACV-ACAM2000, and may also contribute to pathogenicity of VACV-DUKE. The fact that VACV-DUKE contains a full-length copy of a known virulence factor, while not all viruses in the population do (as evidenced by the culture-derived isolate VACV-ACAM2000), suggests the possibility that this gene contributed greater virulence to VACV-DUKE and played a role in its dissemination from the original site of inoculation.

The functions of the ankyrin homolog and the kelch-like gene are unknown, and their contribution to virulence has not been determined in any poxviruses. Kelch is a 50 amino acid domain, often found in a series of repeats, in numerous cellular proteins of diverse functions; the domain is present in multiple poxvirus proteins. The ankyrin repeat is a 33 amino acid domain and is one of the most common protein-protein interaction domains and is found in functionally diverse proteins. Because of the ubiquity of these protein domains, their simple presence does not provide significant clues as to the function or importance of these deleted genes in viral virulence.

Another major difference among these three isolates, is the presence of an additional 6 kb of DNA in the right ITR of VACV-ACAM2000 compared to VACV-DUKE or VACV-CLONE3, which results in a duplication of 12 ORFs. These ORFs are present as a single copy in the left ITR of VACV-DUKE and VACV-CLONE3. Four of these are full length, and the others are gene fragments. The significance of gene duplication in OPVs is not known.

## Conclusion

The genomic sequence VACV-DUKE, a Dryvax^® ^isolate derived from a patient experiencing vaccine related complications, is overall very similar to that of Dryvax^® ^cell culture-derived clonal isolates. While some major genomic differences exist between isolates, it remains to be determined if these differences, or smaller, nucleotide-level differences are responsible for variation in pathogenicity and contribution to vaccine side effects With the sequences of multiple clones from Dryvax^® ^we can begin to appreciate the diversity of the viral population in the smallpox vaccine.

## Methods

### Cells and viruses and DNA sequencing

VACV-DUKE was obtained from the WHO Collaborating Center for Smallpox and other Poxvirus Infections at the Centers for Disease Control and Prevention Atlanta, GA. Virus was identified after two passages on chick chorioallantoic membrane and then cultured in two successive passages in BSC-40 cells in RPMI-1640 supplemented with 2% fetal bovine serum. Standard procedures were used for isolation of viral DNA, and the sequencing strategy that was used has been previously described [20]. The entire VACV-DUKE genome was divided into 19 overlapping fragments. Each fragment was amplified using the Expand-Long Template PCR System (Roche) from genomic DNA, and sequenced using a bank of sequencing primers. The final consensus DNA sequence represented an average 4.17-fold redundancy with each nucleotide position covered by at least one high-quality sequence read in each direction.

### Regions of heterogeneity

Three regions were cloned for sequencing due to heterogeneity at single nucleotide positions in the genome-derived PCR products. In the first, at nucleotide 12344, 2 of 10 clones contained G, and 8 contained A, therefore we report an A in the final genome. The second position with heterogeneity is found in the 3^rd ^copy of DR1 and DR4 in the ITR region at each end of genome: in the left ITR, 2 out of 12 clones lacked the 42^nd ^A and in the right ITR, 10 out of 12 clones lack the 42^nd ^T. To keep the sequence consistent between the left and right ITR, we report no 42^nd ^A/T in the 3^rd ^copy of DR1/DR4 in both ITR in the final sequence.

### Direct repeats

DR1/DR4 in the ITRs is composed of tandem repeats of 70 nt length and was too long to sequence through. To estimate the number of repeats we cloned the region including DR1 and analyzed the size by agarose gel electrophoresis. Based on the size, 19 copies of the repeat are present.

### Assembly and annotation

Assembly of the raw sequence data was performed using the Staden software package on a Linux platform [21]. Raw data was processed using Pregap4 and a consensus sequence was assembled and edited using Gap4 [22-24]. Gaps were closed using primers based on the assembled contigs. An ORF was defined as a continuous stretch of DNA that can be translated into a polypeptide that is initiated by a methione residue and extended for at least 60 residues prior to a termination codon (TGA, TAA or TAG). ARTEMIS [25], Poxvirus Orthologous Clusters database (POCs) [14,26], BLASTP [27] were used to detect and annotate ORFs. For some ORFs, BLASTN and BLASTP searches were carried out at the NCBI website [27]. Viral Genome Organizer (VGO) software [28] was used to analyze the position and arrangement of genes. NAP [29] was used to compare the nucleotide sequence of the fragmented ORF regions of VACV-DUKE against the corresponding longest protein sequence encoded by orthologous chordopoxvirus (ChPV) genes. Base-By-Base (BBB) software [30] was used to calculate the indels and substitutions among VACV-DUKE, CLONE3 and VACV-ACAM2000. Motifscan against Prosite and Pfam collection [31,32] were used to analyze protein structure and features.

### Phylogenetic analysis

Phylogenetic analysis was carried out using an alignment of an 82 kb central region of the genome conserved among all completely sequenced OPVs. JDotter software [33] and VGO, available at the Viral Bioinformatics Resource Center [34,35] were used to generate dotplots between VACV-DUKE and the other OPVs to determine the borders of the corresponding conserved DNA sequences in the different OPVs. ClustalW 1.83 [36] was used to align the sets of DNA and protein sequences followed by manual correction in BBB. Njplot [37] on a Linux platform and Treeview software [38] on a Windows platform were used to construct a phylogenetic tree with bootstrapping.

### Sequence availability

The VACV-DUKE genome sequence has been deposited in GenBank under accession DQ439815 and at [34]. The GenBank accession numbers of the genomes used for comparison are: VACV-Copenhagen: M35027; VACV-MVA: U94848; VACV-CLONE3: AY313848; VACV-ACAM2000: AY313847; VARV-Bangladesh-1975 (BSH): L22579; VARV-India-1967 (IND): X69198; VARV-Garcia-1966 (GAR): Y16780; MPXV-Zaire-96-I-16 (ZAI): AF380138; ECTV-Moscow (MOS): AF012825; CPXV-Brighton Red (BR): AF482758; camelpox virus-M-96: AF438165; ECTV-NAVAL (available at [34]), CMLV-CMS: AY009089, CMLV-M96: NC_003391.1, MPXV-SL: AY741551; VACV-WR: AY243312.1; RPXV-UTR: NC_005858; CPXV-GRI: X94355.

## Competing interests

The author(s) declare that they have no competing interests.

## Authors' contributions

GL assembled and analyzed the sequence data; NC designed the sequencing strategy and assisted in data analysis; ZF performed the sequencing reactions, RMLB designed the sequencing strategy and assisted in data analysis; JO sequenced VACV-ACAMB2000 and VACV-Clone3; TH provided the isolate and prepared the template; ID provided the isolate and prepared the template; CU assisted in analysis, assembly and writing of the manuscript; DJE wrote the manuscript and assisted in interpretation of results.

## Supplementary Material

Additional file 1 Predicted genes in VACV-DUKE. Table of predicted genes in VACV-DUKE with relationships to orthologs in VACV-CLONE3 and VACV-ACAM2000. Click here for file
