# Supplementary material for: Genomic sequence and analysis of a vaccinia virus isolate from a patient with a smallpox vaccine-related complication
Source: Virol J. 2006 Oct 25;3:88. doi: 10.1186/1743-422X-3-88 (PMC1635044; doi:10.1186/1743-422X-3-88)
Supplement: Additional file 1 — Predicted genes in VACV-DUKE. Table of predicted genes in VACV-DUKE with relationships to orthologs in VACV-CLONE3 and VACV-ACAM2000. [file 1743-422X-3-88-S1.pdf]

Predicted Function/Motif	VACV-COP	VACV-DUKE					ACAM2000			VACV-CLONE3		
	Ortholog	Fragment	ORF	Length	Start	Stop	ORF	Length	%Ident	ORF	Length	%Ident
Chemokine binding protein	C23L		1	241	2847	3572	1	241	100	1	241	100
	B29R		225	241	196389	197114	241	241	100	241	241	100
TNF-alpha receptor	n/a	yes	2	146	3976	4416	2 [1]	146	100	2 [1]	146	100
	n/a	yes	224	146	195545	195985	240 [1]	146	100	240 [1]	146	100
	C22L	yes	3	122	4299	4667	3 [1]	122	99.18	3 [1]	122	99.18
	B28R	yes	223	122	195294	195662	239 [1]	122	99.18	239 [1]	122	99.18
Ankyrin (Cop-B25R)	C21L	yes	4	111	5031	5366	4 [1]	111	100	4 [1]	111	100
	B27R	yes	222	111	194595	194930	238 [1]	111	100	238 [1]	111	100
	C20L	yes	5	77	5299	5532	5 [1]	128	100	5 [1]	77	100
	B26R	yes	221	77	194429	194662	237 [1]	128	100	237 [1]	77	100
	C19L	yes	6	146	5935	6375	6 [1]	137	90.41	6 [1]	149	97.99
	B25R	yes	220	146	193586	194026	236 [1]	137	90.41	236 [1]	149	97.99
Ankyrin (CPXV-008)	C18L	yes	7	198	6617	7213	7 [1]	198	100	7 [1]	198	99.49
	B24R	yes	219	198	192748	193344	235 [1]	198	100	235 [1]	198	99.49
	C17L	yes	8	382	7307	8455	8 [1]	382	99.74	8 [1]	382	99.21
	B23R	yes	218	382	191506	192654	234 [1]	382	99.74	234 [1]	382	99.21
Unknown (Cop-B22R)	C16L		9	181	8502	9047	9	181	100	9	181	100
	B22R		217	181	190914	191459	233	181	100	233	181	100
Unknown (Cop-C15L)	C15L	yes	10	91	9383	9658	10	89	91.21	10	91	100
	B21R	yes	216	91	190303	190578	232	89	91.21	232	91	100
Surface glycoprotein	n/a	yes	11	51	10069	10224	11 [1]	51	100	11 [1]	51	100
	n/a	yes	215	51	189737	189892	231 [1]	51	100	231 [1]	51	100
Unknown (Cop-C14L)	C14L		12	190	10476	11048	12 [1]	190	100	12 [1]	190	99.47
	n/a		214	190	188913	189485	232 [1]	190	100	232 [1]	190	99.47
Serpin (SPI)	C12L		13	309	11210	12139	13	357	100	13	334	97.76
	n/a		213	357	187678	188751	229	357	100	229	353	98.32
Growth factor	C11R		14	141	12706	13131	14/228	139	97.87	14	140	97.87
IL-1 Receptor antagonist	C10L		15	331	13286	14281	15/227	331	98.79	15	331	99.7
RING finger/apoptosis	n/a	yes	16	44	14794	14928	16 [1]	44	100	16 [1]	44	100
	n/a	yes	17	83	14889	15140	17/226 [1]	83	97.59	17 [1]	83	97.59
	n/a	yes	18	62	15285	15473	18/225 [1]	62	98.39	18 [1]	62	96.77
IL-18 BP (C12L)	n/a		19	124	15646	16020	19/224	124	95.16	19	124	89.52
Host Range (Bang-D8L)	n/a	yes	20	90	16079	16351	20/223 [1]	90	100	20 [1]	90	100
	n/a	yes	21	142	16364	16792	21/222 [1]	142	99.3	21 [1]	142	99.3
	n/a	yes	22	135	16881	17288	22/221 [1]	135	99.26	22 [1]	135	99.26
	n/a	yes	23	77	17537	17770	23/220 [1]	77	100	23 [1]	77	100
	n/a	yes	24	71	17792	18007	24/219 [1]	71	98.59	24 [1]	71	98.59
Retroviral pseudoprotease (ORFV)	n/a		25	55	18111	18278	25/218	55	100	25	56	98.21
Ankyrin (Cop-C9L)	C9L		26	634	18451	20355	26	634	99.68	26	634	100
							217 [1]	91				
Unknown (Cop-C8L)	C8L		27	177	20398	20931	27	177	100	27	177	93.22
Host range virulence factor	C7L		28	150	21003	21455	28	150	100	28	150	100
Unknown (Cop-C6L)	C6L		29	151	21682	22137	29	151	100	29	151	100
Unknown (Cop-C5L)	C5L		30	201	22256	22861	30	204	100	30	204	100
IL-1 Receptor antagonist	C4L	yes	31	62	22933	23121	31 [1]	62	98.39	31 [1]	62	98.39
	C4L	yes	32	76	23108	23338	32 [1]	136	94.74	32 [1]	136	94.74
	C4L	yes	33	59	23662	23841	33 [1]	59	98.31	33 [1]	59	96.61
Complement binding (secreted)	C3L		34	263	23908	24699	34	263	99.24	34	261	98.48

Predicted Function/Motif	VACV-COP	VACV-DUKE					ACAM2000			VACV-CLONE3		
	Ortholog	Fragment	ORF	Length	Start	Stop	ORF	Length	%Ident	ORF	Length	%Ident
Kelch-like (Cop-C2L)	C2L		35	506	24766	26286	35	506	99.8	35	506	99.8
Unknown (Cop-C1L)	C1L		36	224	26356	27030	36	224	100	36	224	99.55
Virokine (Cop-N1L)	N1L		37	117	27017	27370	37	117	100	37	117	100
Alpha-amanitin sensitivity	N2L		38	175	27489	28016	38	175	98.86	38	175	99.43
Ankyrin (Cop-M1L)	M1L		39	469	28058	29467	41	469	100	39 [1]	72	100
			39	469	28058	29467	41	469	100	40 [1]	214	99.51
			39	469	28058	29467	41	469	100	41 [1]	159	97.00
Unknown (Cop-M2L)	M2L		40	220	29445	30107	42	220	99.09	42	220	99.09
Ankyrin/Host range (Cop-K1L)	K1L		41	284	30242	31096	43	284	100	43	284	100
Serpin (SPI)	K2L		42	369	31318	32427	44	369	100	44	369	99.73
INF resistance (PKR inhibitor)	K3L		43	88	32477	32743	45	88	100	45	88	100
Phospholipase D-like (Cop-K4L)	K4L		44	424	32795	34069	46	424	100	46	424	100
Putative monoglyceride lipase	K5L	yes	45	171	34096	34611	47 [1]	136	99.06	47 [1]	121	99.06
	K6L	yes	46	81	34633	34878	48 [1]	81	100	48 [1]	110	100
Unknown (Cop-K7R)	K7R		47	149	35017	35466	49	149	100	49	149	100
Apoptosis inhibitor (mitochondrial-associated)	F1L		48	226	35531	36211	50	226	99.56	50	226	99.56
dUTPase	F2L		49	147	36223	36666	51	147	100	51	147	100
Kelch-like (Cop-F3L)	F3L		50	480	36690	38132	52	480	100	52	480	99.79
Ribonucleotide Reductase small subunit	F4L		51	319	38143	39102	53	319	100	53	319	100
36kDa major membrane protein (Cop-F5L)	F5L		52	321	39134	40099	54	321	99.38	54	321	99.69
Unknown (Cop-F6L)	F6L		53	74	40129	40353	55	74	100	55	74	100
Unknown (Cop-F7L)	F7L		54	80	40369	40611	56	80	100	56	80	100
Cytoplasmic protein (Cop-F8L)	F8L		55	65	40755	40952	57	65	98.46	57	65	100
Putative MP (Cop-F9L)	F9L		56	212	41012	41650	58	212	100	58	212	99.53
Ser/Thr kinase Morph (Cop-F10L)	F10L		57	439	41637	42956	59	439	99.77	59	439	99.77
Unknown (Cop-F11L)	F11L		58	348	42979	44025	60	348	100	60	348	100
Actin tail/microtubule	F12L		59	635	44068	45975	61	635	99.69	61	635	99.37
Phospholipase EEV (Cop-F13L)	F13L		60	372	46009	47127	62	372	100	62	372	100
Unknown (Cop-F14L)	F14L		61	73	47145	47366	63	73	100	63	73	98.63
Unknown (Cop-F15L)	F15L		62	158	47638	48114	64	158	99.37	64	158	99.37
Unknown (Cop-F16L)	F16L		63	231	48121	48816	65	231	99.13	65	231	100
DNA-binding phosphoprotein (Cop-F17R)	F17R		64	101	48879	49184	66	101	99.01	66	101	100
Poly (A) polymerase-large (VP55)	E1L		65	479	49181	50620	67	479	99.79	67	479	100
Unknown (Cop-E2L)	E2L		66	737	50617	52830	68	737	99.86	68	737	100
IFN resistance/PKR inhibitor (Z-DNA binding)	E3L		67	190	52931	53503	69	190	100	69	190	96.84
RNA pol (RPO30)	E4L		68	259	53559	54338	70	259	100	70	259	100
Virosome component	E5R		69	331	54415	55410	71 [1]	189	99.46	71 [1]	189	99.46
							72 [1]	149	99.46	72 [1]	149	99.46
Unknown (Cop-E6R)	E6R		70	567	55547	57250	73	567	99.82	73	567	99.29
Soluble/Myristyl EEV (Cop-E7R)	E7R		71	166	57332	57832	74	166	100	74	166	100
ER-localized MP(Cop-E8R)	E8R		72	273	57957	58778	75	273	100	75	273	100
DNA pol	E9L		73	1005	58785	61802	76	1005	99.8	76	1005	100
Redox/EVR-1 Morph	E10R		74	95	61834	62121	77	95	100	77	95	100

Predicted Function/Motif	VACV-COP	VACV-DUKE					ACAM2000			VACV-CLONE3		
	Ortholog	Fragment	ORF	Length	Start	Stop	ORF	Length	%Ident	ORF	Length	%Ident
Virion core ES	E11L		75	129	62116	62505	78	129	100	78	129	100
Unknown (Cop-O1L)	O1L		76	666	62492	64492	79	666	99.55	79	666	99.85
Glutaredoxin 1 (Cop-O2L)	O2L		77	108	64532	64858	80	108	100	80	108	100
DNA-binding protein (Cop-I1L)	I1L		78	312	65004	65942	81	312	99.68	81	312	99.36
Unknown (Cop-I2L)	I2L		79	73	65949	66170	82	73	95.89	82	73	97.26
DNA-binding phosphoprotein (Cop-I3L)	I3L		80	269	66171	66980	83	269	100	83	269	100
Ribonucleotide Reductase large subunit	I4L		81	771	67063	69378	84	771	100	84	771	99.87
IMV protein VP13	I5L		82	79	69405	69644	85	79	100	85	79	100
Telomere-Binding protein	I6L		83	382	69663	70811	86	382	99.74	86	382	99.74
Virion Core Protease	I7L		84	423	70804	72075	87	423	100	87	423	100
RNA helicase/NPH-II	I8R		85	676	72081	74111	88	676	99.7	88	676	99.85
Predicted metallo-protease (Cop-G1L)	G1L		86	591	74115	75890	89	591	100	89	591	100
VLTF (late transcription elongation factor - Cop-G2R)	G2R		87	220	76216	76878	91	220	99.09	91	220	99.09
Unknown (Cop-G3L)	G3L		88	111	75887	76222	90	111	100	90	111	100
Glutaredoxin 2 (Cop-G4L)	G4R		89	124	76848	77222	92	124	100	92	124	100
Unknown (Cop-G5R)	G5R		90	434	77225	78529	93	434	98.62	93	434	98.62
RNA pol (RPO7)	G5.5R		91	63	78537	78728	94	63	100	94	63	100
Unknown (Cop-G6R)	G6R		92	165	78730	79227	95	165	97.58	95	165	96.97
Structural protein (Cop-G7L)	G7L		93	371	79192	80307	96	371	100	96	371	100
VLTF-1 (Cop-G8R)	G8R		94	260	80338	81120	97	260	100	97	260	100
Myristylated protein (Cop-G9R)	G9R		95	340	81140	82162	98	340	100	98	340	100
Myristylated MP IMV (Cop-L1R)	L1R		96	250	82163	82915	99	250	100	99	250	100
Unknown (Cop-L2R)	L2R		97	85	82947	83204	100	85	100	100	85	100
Unknown (Cop-L3L)	L3L		98	350	83194	84246	101	350	100	101	350	100
Core package/transcription	L4R		99	251	84271	85026	102	251	100	102	251	99.6
Putative MP (Cop-L5R)	L5R		100	128	85036	85422	103	128	100	103	128	100
Virion (Cop-J1R)	J1R		101	153	85379	85840	104	153	98.69	104	153	98.69
Thymidine kinase	J2R		102	177	85856	86389	105	177	100	105	177	100
Poly(A) polymerase-small (VP39)	J3R		103	333	86455	87456	106	333	100	106	333	100
RNA pol (RPO22)	J4R		104	185	87371	87928	107	185	100	107	185	100
Late Es MP (Cop-J5L)	J5L		105	133	87990	88391	108	133	100	108	133	100
RNA pol (RPO147)	J6R		106	1286	88498	92358	109	1286	99.92	109	1286	99.92
Tyr/Ser phosphatase	H1L		107	171	92355	92870	110	171	100	110	171	100
component of fusion complex	H2R		108	189	92884	93453	111	189	100	111	189	100
IMV morphogenesis	H3L		109	324	93456	94430	112	324	100	112	318	98.15
RAP94 (RNA pol assoc protein)	H4L		110	795	94431	96818	113	795	99.87	113	795	99.87
VLTF-4 (late transcription factor 4)	H5R		111	203	97004	97615	114	203	100	114	203	100
Topoisomerase type I	H6R		112	314	97616	98560	115	314	100	115	314	100
Unknown (Cop-H7R)	H7R		113	146	98597	99037	116	146	97.95	116	146	98.63
Large capping enzyme	D1R		114	844	99081	101615	117	844	99.88	117	844	100
Virion Core (Cop-D2L)	D2L		115	146	101574	102014	118	146	100	118	146	100
Virion core (Cop-D3R)	D3R		116	237	102007	102720	119	237	100	119	237	99.58
Uracil-DNA glycosylase	D4R		117	218	102720	103376	120	218	100	120	218	100
NTPase, DNA replication	D5R		118	785	103408	105765	121	785	100	121	785	100
Morph, VETF-s (early transcription factor small)	D6R		119	637	105806	107719	122	637	100	122	637	100

Predicted Function/Motif	VACV-COP	VACV-DUKE					ACAM2000			VACV-CLONE3		
	Ortholog	Fragment	ORF	Length	Start	Stop	ORF	Length	%Ident	ORF	Length	%Ident
RNA pol 18(RPO18)	D7R		120	161	107746	108231	123	161	100	123	161	100
Carbonic anhydrase/Virion	D8L		121	304	108194	109108	124	304	99.34	124	304	99.34
mutT motif/NTP-PPH	D9R		122	213	109150	109791	125	213	100	125	213	100
mutT motif/NPH-PPH/down regulator	D10R		123	248	109788	110534	126	248	100	126	248	100
NPH-I, virion	D11L		124	631	110535	112430	127	631	100	127	631	100
Small capping enzyme	D12L		125	287	112465	113328	128	287	100	128	287	100
Rifampicin resistance MP	D13L		126	551	113359	115014	129	551	100	129	551	100
VLTF-2 (late transcription factor 2)	A1L		127	150	115038	115490	130	150	100	130	150	100
VLTF-3 (late transcription factor 3)	A2L		128	224	115511	116185	131	224	100	131	224	100
Thioredoxin-like (Cop-A2.5L)	A2.5L		129	76	116182	116412	132	76	100	132	76	100
P4b precursor	A3L		130	644	116427	118361	133	644	99.22	133	644	99.38
Core protein (Cop-A4L)	A4L		131	281	118414	119259	134	281	100	134	281	100
RNA pol 19 (RPO19)	A5R		132	164	119297	119791	135	164	100	135	164	100
Unknown (Cop-A6L)	A6L		133	372	119788	120906	136	372	100	136	372	99.46
VETF-L (early transcription factor large)	A7L		134	710	120930	123062	137	710	99.72	137	710	99.86
VITF-3 (intermediate transcription factor 3-Cop-A8R)	A8R		135	288	123116	123982	138	288	100	138	288	100
Membrane protein (Cop-A9L)	A9L		136	108	123975	124301	139	108	100	139	108	100
P4a precursor	A10L		137	891	124302	126977	140	891	99.89	140	891	99.55
Unknown (Cop-A11R)	A11R		138	318	126992	127948	141	318	100	141	318	100
Structural protein (Cop-A12L)	A12L		139	191	127950	128525	142	192	97.4	142	191	98.95
Virion MP (Cop-A13L)	A13L		140	68	128549	128755	143	70	95.71	143	70	97.14
IMV PO4 MP (Cop-A14L)	A14L		141	90	128863	129135	144	90	98.89	144	90	98.89
IMV-MP/Virulence factor (Cop-A14.5L)	A14.5L		142	53	129152	129313	145	53	98.11	145	53	98.11
Unknown (Cop-A15L)	A15L		143	94	129303	129587	146	94	100	146	94	100
Soluble/Myristylated (Cop-A16L)	A16L		144	377	129571	130704	147	378	99.21	147	378	99.47
IMV MP PO4 (Cop-A17L)	A17L		145	203	130707	131318	148	203	100	148	203	100
DNA Helicase, transcription	A18R		146	493	131333	132814	149	493	99.8	149	493	100
Unknown (Cop-A19L)	A19L		147	77	132795	133028	150	77	100	150	77	100
DNA Processivity factor	A20R		148	426	133381	134661	152	426	99.53	152	426	99.77
Unknown (Cop-A21L)	A21L		149	117	133029	133382	151	117	100	151	117	99.15
Resolvase	A22R		150	176	134624	135154	153	187	100	153	176	100
VITF-3 (intermediate transcription factor 3, Cop-A23R)	A23R		151	382	135174	136322	154	382	100	154	382	100
RNA pol 132(RPO132)	A24R		152	1164	136319	139813	155	1164	99.74	155	1164	99.83
CPV-ATI protein (Bang-A27L)	A25L	yes	153	65	139818	140015	156 [1]	65	100	156 [1]	65	100
CPV-ATI protein (Bang-A27L)	A26L[2]	yes	154	154	139984	140448	157 [1]	154	100	157 [1]	154	100
CPV-ATI protein (Bang-A27L)	n/a	yes	155	227	140720	141403	158 [1]	227	100	158 [1]	227	100
CPV-ATI protein (Bang-A27L)	n/a	yes	156	721	141363	143528	159 [1]	721	99.86	159 [1]	721	99.86
IMV surface protein/ATI factor	A26L		157	500	143573	145075	160	500	99.2	160	500	99.4
Fusion protein (Cop-A27L)	A27L		158	110	145125	145457	161	110	100	161	110	100
IMV MP/Virus entry (Cop-A28L)	A28L		159	146	145458	145898	162	146	100	162	146	100
RNA pol 35(RPO35)	A29L		160	305	145899	146816	163	305	99.67	163	305	99.67

Predicted Function/Motif	VACV-COP	VACV-DUKE					ACAM2000			VACV-CLONE3		
	Ortholog	Fragment	ORF	Length	Start	Stop	ORF	Length	%Ident	ORF	Length	%Ident
Virion Morph (Cop-A30L)	A30L		161	77	146779	147012	164	77	100	164	77	100
Unknown (Cop-A31R)	A31R		162	124	147172	147546	165	124	100	165	124	100
ATPase/DNA packaging protein	A32L		163	270	147513	148325	166	270	99.63	166	270	99.63
EEV Glycoprotein (Cop-A33R)	A33R		164	185	148443	149000	167	185	100	167	185	100
EEV Glycoprotein (Cop-A34R)	A34R		165	168	149024	149530	168	168	99.4	168	168	100
Unknown (Cop-A35R)	A35R		166	176	149574	150104	169	176	100	169	176	100
IEV-specific (Cop-A36R)	A36R		167	221	150171	150836	170	221	100	170	221	100
Unknown (Cop-A37R)	A37R		168	263	150903	151694	171	263	99.62	171	263	99.24
Unknown (Gar-A43R)	n/a		169	62	151784	151972	172	62	100	172	62	100
CD47-like	A38L		170	277	151969	152802	173	277	100	173	277	100
Semaphorin	A39R	yes	171	228	152819	153505	174 [1]	132	96.27	174 [1]	134	99.25
		yes	172	142	153589	154017	175 [1]	142	95.77	175 [1]	142	99.3
Lectin homolog	A40R		173	168	154043	154549	176	168	98.21	176	168	99.4
Unknown (Cop-A41L)	A41L		174	219	154607	155266	177	219	99.09	177	219	100
Profilin homolog (Cop-A42R)	A42R		175	133	155431	155832	178	133	100	178	133	100
Membrane glycoprotein-class I	A43R		176	196	155870	156460	179	194	96.43	179	194	96.43
Unknown (MVA-156R)	n/a		177	78	156468	156704	n/a	n/a	n/a	180	78	100
Hydroxysteroid dehydrogenase	A44L		178	346	156805	157845	181	346	99.42	181	346	99.42
Superoxide dismutase-like	A45R		179	125	157892	158269	182	125	99.2	182	125	100
IL-1 signaling inhibitor (Cop-A46R)	A46R		180	240	158259	158981	183	240	100	183	240	100
Unknown (Cop-A47L)	A47L		181	244	159069	159803	184	244	99.59	184	244	99.18
Thymidylate kinase	A48R		182	227	159834	160517	185	204	100	185	227	100
Unknown (Cop-A49R)	A49R		183	162	160541	161029	186	162	100	186	162	98.77
DNA ligase	A50R		184	552	161061	162719	187	552	99.46	187	552	99.64
Unknown (Cop-A51R)	A51R	yes	185	69	162765	162974	188 [1]	69	100	188	330	99.62
		yes	186	266	162953	163753	188.1 [1]	266	99.62			
Intracellular TLR and IL-1 signaling inhibitor (Cop-A52R)	A52R		187	190	163823	164395	189	190	98.95	189	190	100
TNF-alpha receptor	A53R		188	185	164716	165273	190 [1]	132	96.21	190	186	96.77
Kelch-like (Cop-A55R)	A55R		189	564	165528	167222	191	564	99.11	191	564	99.29
Hemagglutinin	A56R		190	309	167272	168201	192	309	99.03	192	309	99.03
Guanylate kinase	A57R		191	151	168346	168801	193	151	100	193	151	100
Ser/Thr Kinase (Cop-B1R)	B1R		192	300	168952	169854	194	300	100	194	300	100
Unknown (Cop-B2R)	B2R	yes	193	219	169944	170603	195 [1]	219	100	195 [1]	219	99.09
Unknown (Cop-B2R)	B3R	yes	194	266	170639	171439	196 [1]	124	99.19	196 [1]	264	98.12
Ankyrin (Cop-B4R)	B4R		195	558	171669	173345	197	558	99.64	197	558	99.46
Complement control/CD46/EEV	B5R		196	317	173448	174401	198	317	99.37	198	317	97.79
Unknown (Cop-B6R)	B6R		197	173	174483	175004	199	173	100	199	173	100
Virulence, ER resident	B7R		198	182	175042	175590	200	182	100	200	182	100
IFN-gamma receptor	B8R		199	272	175645	176463	201	272	99.63	201	272	99.63
Virulence factor (Cop-B9R)	B9R	yes	200	77	176550	176783	202 [1]	77	100	202 [1]	77	100
Kelch-like (CPV-GRI-B9R)	B10R	yes	201	166	176746	177246	203 [1]	166	100	203 [1]	166	100
Unknown (Cop-11R)	B11R		202	91	177327	177602	204	72	98.59	204	92	98.9
Ser/Thr Kinase (Cop-B12R)	B12R		203	283	177669	178520	205	283	100	205	283	99.65
Serpin (SPI)	B13R	yes	204	126	178619	178999	206 [1]	126	100	206 [1]	126	100
	B14R	yes	205	222	178974	179642	207 [1]	222	97.3	207 [1]	222	98.2
Unknown (Cop-B22R)	B15R		206	149	179717	180166	208	149	100	208	149	98.66

Predicted Function/Motif	VACV-COP	VACV-DUKE					ACAM2000			VACV-CLONE3		
	Ortholog	Fragment	ORF	Length	Start	Stop	ORF	Length	%Ident	ORF	Length	%Ident
IL-1 beta receptor	B16R		207	326	180250	181230	209	326	98.77	209	326	97.55
Unknown (Cop-B17L)	B17L		208	340	181276	182298	210	340	98.53	210	340	98.82
Ankyrin (Cop-B18R)	B18R		209	574	182438	184162	211	574	98.61	211	574	99.83
IFN-alpha/beta receptor	B19R		210	351	184234	185289	212 [1]	265	97.66	212	353	99.15
Ankyrin (Bang-B18R)	B20R	yes	211	549	185361	187010	n/a			213	791	97.43
kelch-like (EV-M-167)	n/a		n/a				n/a			214	80	-
	n/a		n/a				n/a			215	314	-
	n/a	yes	212	134	187022	187426	n/a			216	134	100

[1] Fragment in ACAM2000 or CLONE3 genomes. Fragments are defined as being truncated by one-third or the ORF is split into two or more pieces.

[2] The first 192 aa of COP-A26L is a fragment of IMV surface protein/ATI factor; aa 193 to 322 COP-A26L is a fragment of CPV-ATI protein (Bang-A27L)

n/a: no ORF in the corresponding region
